# The First Patient with Tibial Hemimelia-Polysyndactyly-Triphalangeal Thumb Syndrome Caused by De Novo c.423+4916 T>C ZRS Variant: A Case Report

**DOI:** 10.3390/ijms25179348

**Published:** 2024-08-29

**Authors:** Paola Montserrat Zepeda-Olmos, Kiabeth Robles-Espinoza, Eduardo Esparza-García, María Teresa Magaña-Torres

**Affiliations:** 1 División de Genética, Centro de Investigación Biomédica de Occidente, Instituto Mexicano del Seguro Social, Sierra Mojada 800, Independencia Oriente, Guadalajara 44340, Jalisco, Mexico; paola.zolmos@alumnos.udg.mx (P.M.Z.-O.); kiabethre@gmail.com (K.R.-E.); 2 Doctorado en Genética Humana, Centro Universitario de Ciencias de la Salud, Universidad de Guadalajara, Sierra Mojada 950, Independencia Oriente, Guadalajara 44340, Jalisco, Mexico; 3 Unidad Médica de Alta Especialidad, Hospital de Pediatría del Centro Médico Nacional de Occidente, Instituto Mexicano del Seguro Social, Belisario Domínguez 735, La Perla, Guadalajara 44360, Jalisco, Mexico; eduardoesparzagenetica@gmail.com

**Keywords:** skeletal disorders, ZRS, *SHH*, limb development, tibial hemimelia-polysyndactyly-triphalangeal thumb syndrome (THPTTS)

## Abstract

Genetic variants in the zone of polarizing activity regulatory sequence (ZRS) that induce ectopic expression of the *SHH* gene have been associated with different ZRS-related phenotypes. We report the first patient with a de novo variant, c.423+4916 T>C, in ZRS (previously classified as a variant of uncertain significance) that causes tibial hemimelia-polysyndactyly-triphalangeal thumb syndrome (THPTTS). A two-month-old male patient presented with bilateral preaxial polydactyly, triphalangeal thumb, and tibial agenesis and was heterozygous for the variant c.423+4916T>C (neither of his parents was a carrier). The findings obtained from the family study were sufficient to reclassify the variant from “uncertain significance” to “likely pathogenic” according to three criteria from the American College of Medical Genetics and Genomics guidelines, as follows: (1) absence of gnomAD, (2) confirmation of paternity and maternity, and (3) strong phenotype–genotype association. In ZRS-associated syndromes, a wide clinical spectrum has been observed, ranging from polydactyly to THPTTS; our patient has the most severe and rare phenotype. We did not perform functional assays. However, the c.423+4916T>C variant is flanked by three variants, which have been proven not only to cause the phenotype but also to increase the expression of *SHH*. Through all this data gathering, we consider the c.423+4916T>C variant to be causative of THPTTS.

## 1. Introduction

Skeletal disorders conform a group of 771 diseases caused by variants within 552 genes. Advances in next-generation sequencing have been useful in describing the etiology of such diseases, which present great phenotypic heterogeneity. However, normal development of the bones and limbs requires the precise spatio-temporal expression of a group of genes; hence, variants in them can lead to similar phenotypes, making the diagnostic workup for patients complex [1].

Particularly, the Sonic Hedgehog (*SHH*) gene participates in limb development across the antero-posterior axis. Genetic variants in the zone of polarizing activity regulatory sequence (ZRS), which induce ectopic expression of *SHH*, have been associated with a spectrum of different ZRS-related phenotypes, including preaxial polydactyly (PPD), triphalangeal thumbs (TPTs), Werner mesomelic syndrome, and tibial hemimelia-polysyndactyly-triphalangeal thumb syndrome (THPTTS) (OMIM 188740) [2].

ZRS acts as an enhancer of *SHH* and is located within intron 5 of the *LMBR1* gene, 1 Mb upstream of its target gene [3]. In this highly conserved region of 775 bp, point variants, duplications, triplications, and insertions have been detected. Specifically, in the Mexican population, Vander Meer et al. reported two families with the c.423+4915C>T (chr7:156791474) ZRS variant, which causes mainly PPD and TPTs. In total, 58 affected individuals were identified, of whom 57 were heterozygous and 1, homozygous. Of the heterozygous patients, 37 had isolated TPT; 2, PPD; 16, TPT and PPD; and 2, radioulnar synostosis. Nonetheless, the homozygous patient presented Werner mesomelic syndrome, a more severe phenotype than that of the heterozygous patients, suggesting a dosage effect [4].

We report the first patient with a confirmed de novo variant c.423+4916T>C (chr7:156791473) in the ZRS that causes tibial hemimelia-polysyndactyly-triphalangeal thumb syndrome. Moreover, with the findings obtained from the family study, the variant was reclassified from “uncertain significance” to “likely pathogenic”.

## 2. Case Presentation

A two-month-old male patient, who had been diagnosed at 7 weeks of gestation with fibular agenesis, was referred by the orthopedic surgeon to a genetics consultation. The patient was born at a gestational age of 39 weeks with normal weight (2750 g), but, due to lower limb deficiency, he had a reduced length (43 cm). He was the first child of apparently healthy parents with no family history of skeletal disorders. His father was 38 years old and his mother, 36.

**Figure 1:**
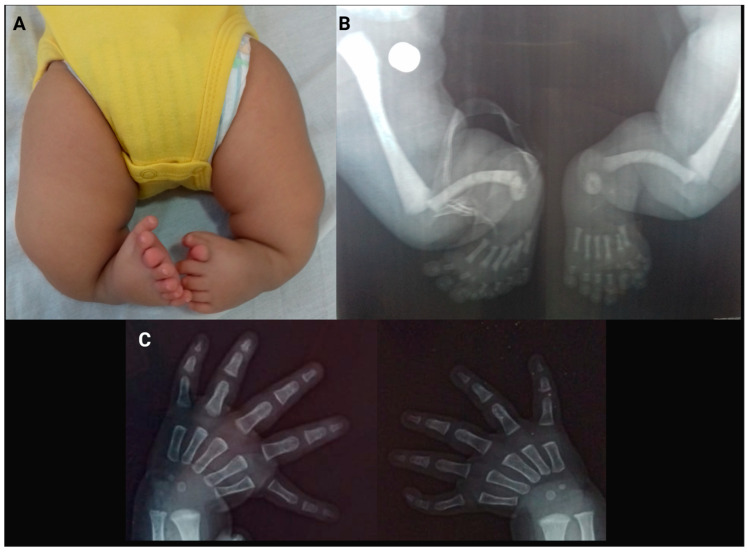
Lower limbs. Photograph (**A**) and X-ray bone radiographic imaging (**B**) showing bilateral tibial agenesis of our patient. Hand X-rays (**C**) showing bilateral triphalangeal thumb. Created with BioRender.com.

At the first genetics consultation, the clinical characteristics detected in the patient were postural plagiocephaly, large anterior fontanelle, frontal bossing, depressed nasal bridge, a slightly narrow thorax, long thumbs with a finger-like appearance, as well as mesomelia and rhizomelia in the lower limbs. Bone radiographic imaging showed bilateral tibial agenesis, preaxial polydactyly, and mirror-image feet (Figure 1); however, hand radiographic imaging was not suitable for evaluation. With these clinical and radiographic findings, a probable diagnosis of a ciliopathy was considered; then, an abdominal ultrasound and new hand X-rays were requested.

At five months of age, the patient had his second genetics consultation. The abdominal ultrasound was normal, and the probable diagnosis was clubfoot with or without deficiency of long bones and/or mirror-image polydactyly, *PITX1*-related. Radiographic imaging of the hands was obtained at the third consultation, showing triphalangeal thumb (Figure 1) and bilateral shortened ulnae; with these results, the diagnosis of THPTTS, caused by variants in the *LMBR1* gene, was considered. Nevertheless, due to limited financial resources in the family, the molecular diagnosis was not performed since it was not freely available in Mexico. 

The patient has an apparently normal intellect, and although his motor development had been impaired due to the skeletal defects, surgery provided adequate thumb opposition function and quality of life improvement. Therefore, his overall prognosis is good.

Six months after the patient received the clinical diagnosis, the molecular analysis was implemented in our institute. Prior to taking a blood sample from both the patient and his parents, an informed consent was signed. Next, genomic DNA was extracted by means of the DTAB-CTAB method, and, using Oligo 6 software, specific primers for analyzing the ZRS region were designed (primer sequences and PCR conditions available upon request). Two fragments amplified by polymerase chain reaction (636 bp and 699 bp) were purified with ExoSAP-IT (Applied Biosystems, Waltham, MA, USA) and then sequenced with the Big Dye Terminator v3.1. (Applied Biosystems) following the specifications from the supplier. Sequencing reactions were purified with columns packed with Sephadex 50G medium. Capillary electrophoresis was carried out in the ABI PRISM 310 (Applied Biosystems) gene analyzer, and the electropherograms were compared with the reference sequence NM_022458.4.

**Figure 2:**
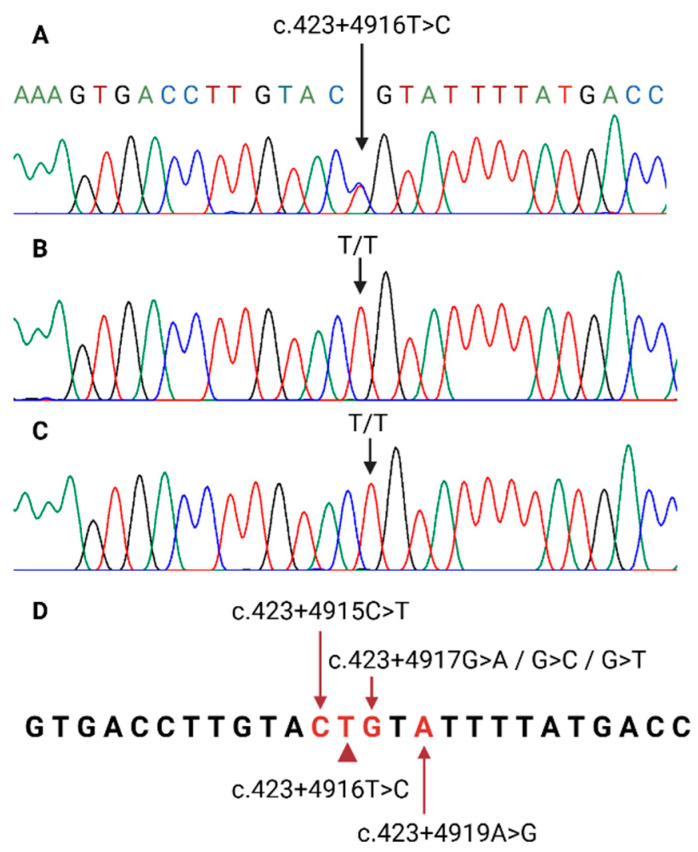
(**A**) Index case electropherogram. The arrow shows the site of variant c.423+4916T>C. (**B**,**C**) show electropherograms of the mother and father. The arrow demonstrates the absence of variation. (**D**) ZRS, region comprising from c.423+4 to c.423+29. The arrowhead shows the c.423+4916T>C variant, surrounded by previously reported pathogenic variants associated with the THPTTS phenotype (arrows). Created with BioRender.com.

In the analysis of the ZRS sequence, the patient was heterozygous for variant c.423+4916T>C (Figure 2), and neither parent had the variant. Further genetic studies of the trio confirmed paternity and maternity (Table 1).

## 3. Discussion

In this report, we described a patient with both clinical and radiographic characteristics of THPTTS and a de novo variant c.423+4916T>C in the ZRS region. He had triphalangeal thumb reconstruction with a good outcome; however, other surgeries, such as the correction of polydactyly and tibial agenesis, are necessary to further improve his quality of life.

In ZRS-associated syndromes, the phenomena of variable expression (inter- and intra-familial), anticipation, and incomplete penetrance have been reported [2,4,5,6]; however, we were not able to identify them in our family since only one individual is affected. These phenomena have been observed in families that harbor three variants in proximity to the one reported in our index case (Figure 2), as follows:

(1) Variant c.423+4915C>T was detected in two Mexican families with a clear autosomal dominant inheritance pattern. In the first family, 46 affected individuals were identified: 1 homozygous and 45 heterozygotes. All patients had TPT, only 30.4% of them had PPD, and, in addition to these two characteristics, the homozygous patient also presented radioulnar synostosis and hypoplastic tibias, evidencing a more severe phenotype. In the second family, 12 heterozygous patients were detected, and their manifestations were heterogeneous: TPT 66.6%, PPD 33.3%, and radioulnar synostosis 16.6% [4].

(2) At position c.423+4917 (chr7:156791472), three different variants have been reported (G>C>A>T), which could highlight it as a hot spot. The G>A transition has been described in Cuban, Korean, Thai, and Turkish families, whereas the G>C and G>T transversions were found in Brazilian and Indian families, respectively [2,6,7]. The patients studied in those investigations, carriers of one of the three variants, presented four main features: TPT (83.3%), PPD (79.1%) (with involvement of hands and feet), and tibial hypoplasia (12.5%); only one patient had tibial aplasia [2,6,7]. 

(3) Variant c.423+4919A>G (chr7:156791470) was described in a family with three heterozygous. The clinical findings were TPT (100%), tibial aplasia (100%: bilateral 66.7% and unilateral 33.3%), and polydactyly in feet (100%). The authors detected not only variable expression but also an increase in severity in the three generations studied [2].

Our patient, heterozygous for the variant c.423+4916 T>C, presented two common characteristics observed in patients with ZRS-related phenotypes: TPTs (a finding that was useful in defining his diagnosis) and PPD (Figure 1). However, he also had bilateral tibial aplasia, which is a rare feature and results in a severe phenotype. This affectation was detected in two patients with the variant c.423+4919 A>G [2]. The clinical variability detected in these patients may be a consequence of the surrounding genomic context and the temporal/spatial activity of other cis-regulatory elements, as has been observed in multiple genetic diseases.

### Classification of Variant c.423+4916T>C

According to the ACMG guidelines, the variant c.423+4916T>C was classified as likely pathogenic with three criteria, as follows: (1) absence of gnomAD (PM2_Supporting), (2) confirmation of paternity and maternity (PS2), and (3) a strong phenotype–genotype association (PPA) [8]. This variant had previously been reported and classified as a VUS in ClinVar [9], and with the findings of this study, it was reclassified as likely pathogenic. Other data that could support the pathogenicity of the c.423+4916 variant and that are not included in the ACMG guidelines are the following: (1) at least three pathogenic variants flanking the position of interest have been described (c.423+4915C>T, c.423+4917G>C/G>A/G>T, and c.423+4917A>G), and their expression studies have suggested that this region is a binding site for transcription factors [2,4,6]; (2) an alignment analysis of the ZRS region with more than 60 species revealed that the segment that includes positions from c.423+4914 to c.423+4924 is 100% conserved.

Although it is well known that variants in the ZRS associated with ectopic SHH expression cause a wide spectrum of limb anomalies [3,10], it remains unclear how increasing the activity of this enhancer alters limb development [11]. In fact, not even the transcription factors that regulate such processes are fully understood. Thus, for each new variant described in the ZRS, functional studies are essential to classify it as pathogenic. Even though these methodologies are complex and not easy to implement in all laboratories, they are necessary to obtain useful information for the development of bioinformatic tools that will allow for the analysis of variants in non-coding regions. It is also important to include in the ACMG guidelines the criteria that will allow professionals to evaluate this type of understudied variants [12].

Unfortunately, this study suffers from an important limitation: it lacks functional assays. These studies are very relevant, not only to classify the variants but also to increase the knowledge about the ZRS element and its binding sequences for transcription factors that regulate the expression of the *SHH* gene.

## 4. Conclusions

Through all this data gathering, we consider that the c.423+4916T>C variant is causative of THPTTS. Here, we described the first patient with a confirmed de novo variant that had been previously reported as a VUS and that, with the findings of this study, was reclassified as likely pathogenic. Even though the degree of classification of the variant will not change the treatment and prognosis of our patient, it does have an impact on the genetic counseling process.

## Figures and Tables

**Table 1:** Case report timeline.

7 October 2016	Prenatal ultrasound: fibular agenesis.
18 August 2017	Referred to the genetics consultation. A probable diagnosis of ciliopathy was considered.
3 November 2017	A normal abdominal ultrasound was provided, so the diagnosis of clubfoot with or without deficiency of long bones and/or mirror-image polydactyly, which is caused by variants in the *PITX1* gene, was suggested.
14 July 2018	With an X-ray study, a clinical diagnosis of tibial hemimelia-polysyndactyly-triphalangeal thumb syndrome, which is caused by variants in the *LMBR1* gene, was reached.
11 March 2019	A heterozygous variant was found in the ZRS (intron 5 of the *LMBR1* gene).
30 January 2020	Genetic testing of the parents showed no variant in the ZRS.

## Data Availability

Data are contained within the article.
